# Managing of Migraine in the Workplaces: Knowledge, Attitudes and Practices of Italian Occupational Physicians

**DOI:** 10.3390/medicina58050686

**Published:** 2022-05-22

**Authors:** Matteo Riccò, Pietro Ferraro, Vincenzo Camisa, Pasquale Di Palma, Giuseppa Minutolo, Silvia Ranzieri, Salvatore Zaffina, Antonio Baldassarre, Vincenzo Restivo

**Affiliations:** 1 Servizio di Prevenzione e Sicurezza Negli Ambienti di Lavoro (SPSAL), AUSL-IRCCS di Reggio Emilia, Via Amendola n.2, I-42122 Reggio Emilia, Italy; 2 Occupational Medicine Unit, Direzione Sanità, Italian Railways’ Infrastructure Division, RFI SpA, I-00161 Rome, Italy; dott.pietro.ferraro@gmail.com; 3 Health Directorate, Occupational Medicine Unit, Bambino Gesù Children’s Hospital IRCCS, I-00146 Rome, Italy; vincenzo.camisa@opbg.net (V.C.); salvatore.zaffina@opbg.net (S.Z.); 4 Istituto nazionale Assicurazione Infortuni sul Lavoro, INAIL—DM2, Roma Tuscolano, Via Michele de Marco, 20, I-00169 Rome, Italy; p.dipalma@inail.it; 5 Department of Health Promotion Sciences Maternal and Infant Care, Internal Medicine and Medical Specialties “G. D’Alessandro”—Hygiene Section, University of Palermo, I-90127 Palermo, Italy; giuseppa.minutolo@unipa.it (G.M.); vincenzo.restivo@unipa.it (V.R.); 6 Department of Medicine and Surgery, University of Parma, Via Gramsci, 14, I-43126 Parma, Italy; silvia.ranzieri@unipr.it; 7 Occupational Medicine Unit, Careggi University Hospital, I-50134 Florence, Italy; antonio.baldassarre@unifi.it

**Keywords:** migraine, job, occupation, knowledge, attitudes, practices

## Abstract

*Background and Objectives*: Migraine is a debilitating disorder, whose incidence peak in the age group of 30–39 years overlaps with the peak of employment years, potentially representing a significant issue for occupational physicians (OP). The present study was performed in order to characterize their knowledge, attitudes and practices on migraine in the workplaces. *Materials and Methods*: A convenience sample of 242 Italian OP (mean age 47.8 ± 8.8 years, males 67.4%) participated in an internet-based survey by completing a structured questionnaire. *Results*: Adequate general knowledge of migraine was found in the majority of participants. Migraine was identified as a common and severe disorder by the majority of respondents (54.0% and 60.0%). Overall, 61.2% of participants acknowledged migraine as difficult to manage in the workplace, a status that made it more likely for OP understanding its potential frequency (Odds Ratio [OR] 3.672, 95% confidence interval [95%CI] 1.526–8.831), or reported previous managing of complicated cases requiring conditional fitness to work judgement (OR 4.761, 95%CI 1.781–2.726). Moreover, professionals with a qualification in occupational medicine (OR 20.326, 95%CI 2.642–156.358), acknowledging the difficult managing of migraine in the workplaces (OR 2.715, 95%CI 1.034–7.128) and having received any request of medical surveillance for migraine (OR 22.878, 95%CI 4.816–108.683), were more likely to recommend specific requirements for migraineur workers. *Conclusions*: Migraine was recognized as a common disorder, but also as a challenging clinical problem for OP. Participating OP exhibited a substantial understanding of migraine and its triggers, but residual false beliefs and common misunderstanding may impair the proper management of this disorder, requiring improved and specifically targeted interventions.

## 1. Introduction

Migraine is a chronic disorder characterized by recurrent episodes of headache and associated symptoms, including any combination of pain, sensitivity to light, sound and less frequently smell and touch, nausea or vomiting [1,2]. Migraine approximately affects 11% of the adult population of Western countries [1], with a disproportionately greater share of women compared to males (usually ranging from 3:1 to 4:1). In Italy, prevalence has been estimated between 8.2% and 13.7% [3,4,5], accounting for 5.2% of all-cause years lived in disability or YLD [5], while a more recent report from the Global Burden of Disease 2016 Headache Collaboration has identified the worldwide highest age-standardized prevalence rates, ranging from 20% to 21% [6,7].

As the prevalence of migraine usually increases up to 30–39 years of age, gradually decreasing afterwards [1,6,8], it affects the most productive years of an individual’s personal, social and professional development [3,9], overlapping with the peak of the employment years [10]. Unsurprisingly, migraine is therefore recognized as a significant cause of days of work lost and reduced quality of life [6,8,9,10]. In Europe, migraineur workers may lose between 27.6 and 28.8 working days per year, with consequent costs for patients, employers, healthcare systems [3,4]. For example, the total cost of the migraine-related disability in the United States was estimated to exceed USD 13 billion a year in the USA alone, with alleged total costs of lost productivity of around USD 19.6 billion [11,12,13]. More recently, the mean annual cost of migraine in the European Union has been estimated at EUR 1222, including lost productivity (EUR 675) and absenteeism (EUR 371) [14]. However, it should be borne in mind that migraine is not routinely assessed at work, with overall direct and indirect costs that may substantially exceed such estimates [15].

Where legal frameworks have formally implemented their role, occupational physicians (OP) may become key players in the management of migraine in the workplace [14,16,17,18]. According to Italian law, OP are the medical professionals responsible for health promotion in the workplace [19,20], being diffusely involved in the communication of risk, participating in the information and formation of the workers. Moreover, Italian Occupational Health and Safety Legislation requires the OP to participate in the adaptation of workplaces to the requirements of workers, and to inform the workers about the pros and cons of recommended interventions [20,21,22]. Unfortunately, very few data on the actual management of migraine in occupational settings are available.

The main endpoint of this study was to characterize the eventual understanding and key aspects on the managing of migraine by Italian OP by means of their respective knowledge (i.e., the awareness of disease and official recommendations), attitudes (i.e., propensity towards proposed interventions) and practices (i.e., actual implementation of specific interventions, either on the patient or on the workplaces, collectively, KAP). Eventually, our results may be useful for targeting specific informative and educative campaigns dedicated to OP that could, in turn, improve the workers’ health and safety.

## 2. Materials and Methods

### 2.1. Study Design

A cross-sectional questionnaire-based study was performed between 1 May 2020 and 20 May 2020, involving OP to participate in a closed discussion group whose application was officially limited to OP. In total, the group had 2034 unique members, but no information could be obtained regarding how many of these members were active participants.

In order to share the study invitation, the chief researcher contacted the administrators, providing a preventive request of authorization that included a short description of the aims of the survey. Users who clicked on the link were provided with the full study information to give their informed consent to participate to the survey through a direct link (Google Forms; Google LLC; Menlo Park, CA, USA). Only participants who had expressed their consent for study participation were initially inquired through specific dichotomous questions (i.e., Yes vs. No) about the 2 main inclusion criteria: living and working in Italy; being an OP in compliance with the above-mentioned legislation. The inclusion criteria were preventively inquired, and if a potential participant did not to match both inclusion criteria, the survey closed. The survey was anonymous, and no personal data such as name, IP address, email address, or personal information unnecessary to the survey were requested, saved, or tracked. No monetary or other compensation was offered to the participants.

### 2.2. Questionnaire

The questionnaire was specifically designed for this study and elaborated through extensive literature review [1,2,6,8,10,11,12,13,14], and its test–retest reliability was preventively assessed through a survey on 10 OP completing the questionnaire at two different points in time that were ultimately excluded from the final analyses. A correlation coefficient was calculated to compare the two sets of responses: items having a coefficient > 0.80 were interpreted as consistent, and were therefore included in the questionnaire used in this survey. All questions were self-reported, and not externally validated. The final questionnaire included the following sections:

1. Individual characteristics: age, sex, seniority, medical background (i.e., being full specialists in occupational medicine or not; having performed a residency in neurology, psychiatry, or internal medicine); main information sources (i.e., professional courses; medical journals; books; colleagues; new media including wikis, social media, etc.) and the Italian region where the professional mainly worked and lived.
2. Knowledge Test. Knowledge of participants about migraine was assessed by means of a knowledge test containing a set of 11 true/false statements, based on a recent publication from the Italian National Health Institute [23] (e.g., “Typically, one out of 3 women is affected by migraine”; TRUE). A summary score (General Knowledge Score; GKS) was then calculated as follows: when the participants answered correctly, +1 was added to a sum score, whereas a wrong indication or a missing/“don’t know” answer added 0 to the sum score. GKS was then dichotomized by median value in higher vs. lower knowledge status.
3. Risk perception. Participants were initially asked to rate the perceived severity (C^MIG^) and the perceived frequency (F^MIG^) of migraine in Italian adult working population by means of a fully labeled 5-point Likert scale (range: from “not significant” to “very significant”). As perceived risk has been defined as a function of the perceived probability of an event and its expected consequences [24], a Risk Perception Score (RPS) was eventually calculated as follows:

Risk perception = F^MIG^ ∗ C^MIG^

4. Attitudes and Practices. We first inquired participants on the perceived barriers for properly manage migraineur workers, including: the ergonomics of the workplaces, working hours, work rhythms, work-related stress and psychosocial risk factors, characteristics of the workplace, interaction of individual risk factors with work environment. All factors were reported in a full scale of 1 (totally disagree) to 5 (totally agree). Participants were then requested to rate how they perceived the management on the workplace of different disorders, including: migraine, diabetes, asthma, low back pain, work-related upper arm disorders, chronic health disease, fibromyalgia, depression and epilepsy. All the aforementioned disorders were rated 1 (not difficult) to 10 (very difficult), and then arbitrarily dichotomized in low concern (1 to 5) vs. high concern (6 to 10).

Respondents were eventually asked (yes vs. no) whether they had received any previous request of medical surveillance from a migraineur worker, diagnosed any case of migraine in the workers they assisted, and eventually judged any worker conditionally fit, conditionally fit (i.e., requiring one or more of the following measures: avoiding night shifts; avoiding shiftwork; avoiding exposures to extreme temperatures; avoiding exposures to extreme intense lights; avoiding front-office activities; avoiding exposures to irritating chemical agents; increased number/length of pauses), or unfit to work because of migraine. A cumulative score (potential range 0 to 8) was then calculated by adding +1 for any of references to any of the aforementioned requirements and/or unfit judgements.

### 2.3. Ethical Considerations

Before giving their consent to the survey, participants were briefed that the gathered data would be handled anonymously and confidentially. The study had therefore an anonymous, observational design, and did not include clinical data about patients and/or participants. As individual participants cannot be identified based on the presented material, this study caused no plausible harm or stigma to them. A preliminary evaluation by an Ethical Committee was therefore not forcibly required according to the Italian law (Italian Official Journal. 76, dated 31 March 2008).

### 2.4. Data Analysis

Continuous variables were initially tested for normal distribution (D’Agostino and Pearson omnibus normality test): where the corresponding *p*-value was <0.10, normality distribution was assumed as rejected and variables were compared through Mann–Whitney or Kruskal–Wallis test for multiple independent samples. On the other hand, variables passing the normality check (D’Agostino and Pearson *p*-value ≥ 0.10) were compared using Student’s *t*-test or ANOVA, where appropriate. Similarly, association between continuous variables was assessed through calculation of the Pearson’s correlation coefficient or Spearman’s rank correlation coefficient, for variables passing or not passing the normality test. Categorical variables were reported as per cent values, and their distribution in respect of the outcome variable of: (a) having any concern towards the managing of migraine and (b) having reported any conditional requirement for migraineur workers, were initially analyzed through chi-squared test. Internal consistency of the knowledge sections was measured through calculation of the Cronbach’s alpha.

All categorical variables that at univariate analysis were significantly associated (i.e., *p* < 0.05) with outcome variables were included in two distinctive stepwise binary logistic regression analysis models in order to calculate adjusted odds ratios (aOR) and their respective 95% confidence intervals (95%CI). All statistical analyses were performed by means of IBM SPSS Statistics 26.0 for Macintosh (IBM Corp. Armonk, NY, USA).

## 3. Results

### 3.1. Descriptive Analysis

As shown in Table 1, a convenience sample of 242 OP (12.1% of the eligible population) agreed to participating in this study. Among the respondents, most of them were females (67.4%), the mean age was 47.8 years ± 8.8 (35.1% ≥ 50-year-old), and they had seniority as OP of 17.1 years ± 13.7 (76.0% ≥ 10 years). Overall, 45.5% resided in Northern Italy, with 28.5% respondents from Central Italy and 22.7% from Southern Italy. Of them, 88.8% had a full qualification in occupational medicine, while the remaining participants were qualified as OP through their specialization in legal medicine (5.4%) and hygiene and preventive medicine (2.5%). More precisely, 17.2% of participants were involved in the health surveillance of healthcare workers in healthcare settings affiliated with the Italian National Health Service (provincial or even regional-level hospitals).

The majority of respondents reported a previous residency in internal medicine (64.5%), while any residency in neurology and psychiatry was reported by only 14.0% and 4.5% of them, respectively. The most frequently reported information source was identified in professional courses (81.7%), followed by official websites (62.0%), medical journals (51.2%), colleagues (43.8%), books (37.6%), while only 15.3% of them included New Media such as blog, social media, wikis, etc. However, only 8.5% of them reported any previous specific attendance of formation courses on migraine.

### 3.2. Knowledge Test

**Figure 1:**
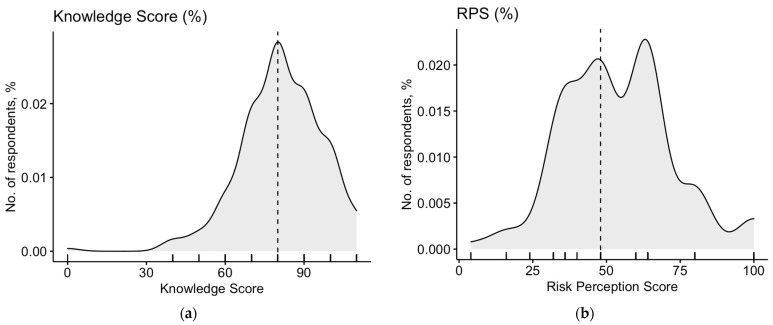
Density plots for: (**a**) General Knowledge Score (GKS) in 242 Italian occupational physicians participating into the survey; (**b**) Density plot Risk Perception Score (RPS). Cumulative scores were substantially skewed (D’Agostino–Pearson’s normality test *p*-value 0.038 and <0.001, respectively). Dotted line represents median value (72.7% and 48.0% for GKS and RPS, respectively).

After normalization, the mean GKS was relatively high (74.0% ± 14.3; actual range, 0.0–100%; median, 72.7%). As shown in Figure 1a, data were skewed (*p* < 0.001), but internal consistency coefficient amounted to Cronbach’s alpha = 0.744, suggesting that the resulting score can be acknowledged as reliable.

In fact (Table 2), nearly all respondents acknowledged the emotional, cognitive and behavioral features of migraine (97.4%), and a large share of them were also aware that the majority of affected cases do not receive appropriate preventive treatment (70.6%), that relapses may last between 4 and 72 h (87.7%), with clinical features different from a pulsating and bilateral headache (64.7%). Moreover, the large majority of participants were aware that stress and hormonal imbalance (97.0%), but also intense noise and bright light (97.9%), can trigger relapses of migraine, while only 36.6% of them had any understanding that extreme temperatures can elicit relapses of migraine. Even though 81.3% of participants understood that females usually exhibit greater presenteeism despite pain and malaise, only 58.7% of participants were aware that up to 1/3 of individuals of female gender may be affected by migraine. Interestingly, 53.2% of respondents correctly acknowledged that the loss of productivity in males is greater than in females, but only 30.2% acknowledged that while females do not have a better quality of life than males. 

### 3.3. Assessment of the Risk Perception

Briefly, 54.0% of participants (No. 127) acknowledged migraine as a common disease, while 60.0% (No. 155) acknowledged its severity as significant or very significant. A corresponding RPS of 54.1% ± 18.7 was calculated (actual range: 4.0–100%, median = 48.0%). As shown in Figure 1b, data were substantially skewed (*p* = 0.038).

### 3.4. Attitudes and Practices

As shown in Table 3, the main barrier for a proper managing of migraineur workers was identified in work-related stress (61.3%), followed by work rhythms (52.8%), working hours (49.4%) and being able to perform appropriate interventions on individuals risk factors for migraine (47.7%). On the contrary, only around one-third of respondents acknowledged as main barriers the ergonomic of the workplace (33.2%), and difficulties in performing appropriate interventions on work-related risk factors.

A total of 159 out of 242 participants (65.7%) reported any previous interaction with migraineur workers, but only 5.0% had previously designed specific medical surveillance programs for migraineur workers. More precisely, 37.9% had previously received any request of medical surveillance from a migraineur worker (35.5% in the previous 5 years, but also 20.7% during the previous calendar year), while 17.8% had diagnosed or contributed to the diagnosis of migraine during their daily practice.

**Figure 2:**
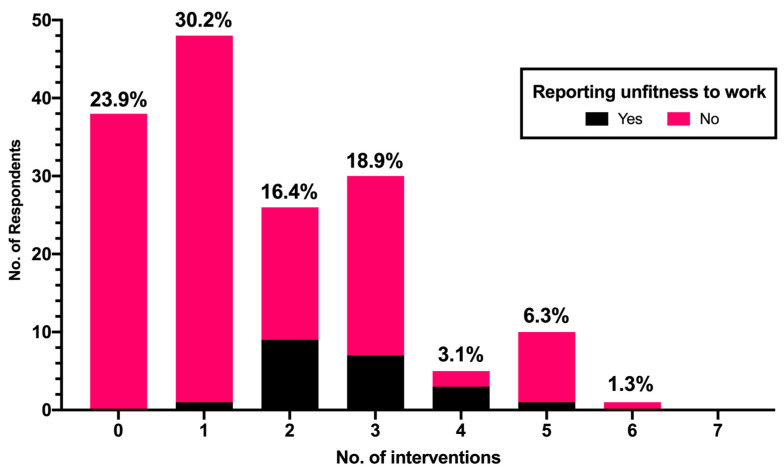
Number of interventions for migraine as reported by participants having any previous interaction with migraineur workers (No. 159 out of 242 total participants).

More than half of participants (54.5%) had previously judged any worker as “conditionally fit” or even unfit (8.3%) because of migraine. Among the main requirements for conditioned fitness, participating OP recalled the prohibition of night shift (28.5%), the implementation of increased number/length of pauses (20.2%), avoiding exposure to extreme and intense lights (20.7%) and temperatures (13.2%), avoiding front-office activities (18.2%), while only 12.4% were banned from shiftwork, and 3.7% required that workers be restricted to potential exposures to irritating chemical agents. An average score of 1.3 (range: 0 to 6) was calculated. Even when the analyses were restricted to medical professionals having a previous experience with migraineur workers (Figure 2), around 23.9% of respondents did not refer any intervention, while 30.2% reported only one intervention (average score 1.7, range: 0 to 6). However, only 7 out of 132 participants formulating a conditional fitness or unfitness judgement had reportedly received any appeal (2.9%).

As reported in Table 4, when participants were asked to rate the perceived difficulties they had in managing migraine in the workplaces, it was associated with an average score of 6.0 ± 2.0, that was similar to the scores for asthma (6.2 ± 1.9, *p* = 0.525) and diabetes (6.3 ± 1.9, *p* = 0.338), but substantially lower than that reported for fibromyalgia (6.9 ± 2.3, *p* < 0.001), low back pain (7.2 ± 2.1, *p* < 0.001), work-related upper arm disorders (7.1 ± 1.9, *p* < 0.001), depression (7.2 ± 1.9, *p* < 0.001), chronic heart disease (7.4 ± 1.6, *p* < 0.001).

### 3.5. Univariate Analysis

No substantial differences were identified in RPS (55.1% ± 18.6 vs. 52.2% ± 18.8) and GKS (74.7% ± 13.4 vs. 72.8% ± 16.0) for professionals having previously managed or not migraineur workers (*p* = 0.135 and *p* = 272, respectively). Additionally, the perceived difficulties in the managing of migraine were similar in professionals having or not previous expertise with migraineur workers (6.01 ± 2.0 vs. 6.12 ± 2.2, *p* = 0.724). In correlation analyses, RPS was positively correlated with GKS (rho = 0.184, *p* = 0.004), while, in turn, GKS was positively associated with the number of interventions required for migraineur workers (rho = 0.129, *p* = 0.045). In other words, a better understanding of migraine was associated with a greater risk perception and more frequent requirements on patients. RPS and the number or requirement were then positively associated with the perceived difficulty in the managing of migraine in the workplace (rho = 0.285, *p* < 0.001 and rho = 0.182, *p* = 0.005, respectively).

As shown in Table 5, medical professionals who acknowledged a greater difficulty in the managing of migraine in the workplaces more frequently identified migraine as a frequent or very frequent disorder (63.5% vs. 39.4%, *p* < 0.001), and a severe or very severe condition (75.7% vs. 50.0%, *p* < 0.001). Moreover, participants with greater concern for migraine management had more frequently judged any worker as conditionally fit to work compared to those with lower concerns (62.8% vs. 41.5%). On the contrary, previous training in neurology was associated with lower concerns (10.5% vs. 20.2%, *p* = 0.037), and similarly reporting colleagues as the main information source on migraine (33.1% vs. 60.6%, *p* < 0.001).

When focusing on the practices reported by participants having had any previous occupational encounter with migraineur workers (*n* = 159; Table 6), having implemented at least one specific intervention for migraine management was positively associated with the specialization in occupational medicine (95.9% vs. 81.6% in those who claimed other background formation, *p* = 0.011) and acknowledging migraine as difficult to manage in the workplaces (68.6% vs. 42.1%, *p* = 0.006). Additionally, reporting any previous request for medical surveillance from migraineur workers (64.5% vs. 15.8%, *p* < 0.001), and having achieved any diagnosis of migraine among assisted workers (26.4% vs. 7.9%, *p* = 0.029) was substantially associated with previous requirements for fitness to work in migraineur workers. On the contrary, working as OP in a hospital affiliated with the National Health Services was negatively associated with the outcome variable (9.1% vs. 34.2%, *p* < 0.001).

### 3.6. Regression Analysis

In regression analyses, the outcome variables of perceiving greater concern for occupational managing of migraine (Model 1; Table 5) and reporting any requirement for conditional fitness to work (Model 2; Table 6) were assessed through two distinctive models that included the following explanatory variables.

Model 1: previous training in neurology; reporting colleagues as priority information sources on migraine; acknowledging migraine as a frequent/very frequent disorder; acknowledging migraine as a severe/very severe disorder; having received any request of medical surveillance from migraineur workers; having achieved any diagnosis of migraine in medical practice; having reported any previous judgement of “conditional fitness” because of migraine; having reported any previous judgement of “unfitness” because of migraine. Acknowledging migraine as a frequent/very frequent condition (aOR 2.730; 95%CI 1.495 to 4.984), and as a severe/very severe disorder (aOR 2.347; 95%CI 1.277 to 4.311), and having reported any previous judgement of “conditional fitness” because of migraine (aOR 4.761, 95%CI 1.781 to 12.726) were identified as positive effectors.

Model 2: specialization in occupational medicine; working as OP in hospital(s) affiliated with the National Health Service; acknowledging migraine as difficult to manage in the workplace; having received any request of medical surveillance from migraineur workers; having diagnosed migraine in medical practice. Additionally, specialization in occupational medicine (aOR 20.326; 95%CI 2.642 to 156.358), acknowledging migraine as difficult to be managed in the workplaces (aOR 2.715; 95%CI 1.034 to 7.128), and reporting any request of medical surveillance by migraineur workers (aOR 22.878; 95%CI 4.816 to 108.683) were acknowledged as positive effectors. On the contrary, working as OP in any hospital(s) affiliated with National Health Service was characterized as a negative effector (aOR 0.036; 95%CI 0.006 to 0.205).

## 4. Discussion

In our cross-sectional study, migraine was recognized as a common issue for the participating 242 OP. As migraine is one of the most common neurological diseases [3,25,26], this was not unexpected. Notwithstanding, a large share of participants exhibited significant uncertainties in terms of its actual burden of disease. From a global perspective, migraine has been classified as the second cause of years lived with disability, and the first one among individuals ≤ 50 years old [25,27,28], but its occurrence and its potential severity were substantially overlooked by study participants, with a resulting unexpectedly low RPS. In this regard, the summary scores were quite skewed, and the risk perception was well correlated with knowledge status as summarized by GKS, i.e., a better understanding of the issue associated with a diagnosis of migraine was associated with a greater risk perception.

Despite a generally high knowledge status among the study participants, the summary GKS was in turn quite skewed: although some features of migraine were acknowledged by the large majority of respondents (e.g., the relevance of noise and bright light in triggering relapses, 97.9%; the emotional and cognitive impacts of migraine, 97.4%; the role of stress and hormonal imbalance in eliciting relapses of migraine, 97.0%) [1,29], some false beliefs were extensively reported. For instance, only half of the participants had any understanding of the substantial prevalence of migraine in individuals of female gender (58.7%) [1,30,31,32], as well as of the greater loss of productivity in males compared to females (53.2%), while only a third of sampled OP understood the limited role of extreme temperatures in eliciting relapses of migraine (36.6%) [1,32,33]. Such misunderstanding about the features of migraine, particularly when dealing with workplace and working-age populations, may substantially impair the proper management of migraineur workers [34,35,36]. At least in the current Italian legal framework, OP are requested to give advice to the employees and employers (health education) and reply to work-related risks by promotion of individual or collective prevention measures [20,37]. Therefore, the sharing by OP of misbeliefs or even false beliefs on the epidemiology and risk factors of migraine may be of certain relevance as potentially detrimental, both for employers and employees, through increased absenteeism and the resulting loss of productivity. More precisely, not acknowledging that, for example, relapses may be triggered by environmental factors such as low and high temperatures [38,39], may prevent OP from evaluating the worker as conditionally fit or even totally unfit for certain tasks. Similarly, failing to properly recognize actual risk factors would result in inaccurate management, and higher relapse rates. In this regard, the relatively low share of participants identifying main risk factors such as working rhythms and working hours as potential barriers to a proper management of migraine may be particularly frightening [34,35,36,40,41,42,43].

In fact, an unexpected outcome of this study was represented by the general underscoring not only of the actual clinical features of migraine, but also of the very complicated management of this disorder in the workplaces [2,34,35,36]. When participants were asked to rank migraine compared to other very common conditions, it was identified as the least difficult to manage by the perspective of OP, being substantially outscored by chronic heart disease, low back pain, depression, upper arm disorders, epilepsy, but also fibromyalgia. As RPS was positively associated with the perceived difficulty in the management of migraine, it is reasonable that a main driver of the low-risk perception was in fact represented by the lack of personal expertise. Only half of the respondents had any previous encounters with migraineur workers, leaving them potentially unable to identify how heterogenous and therefore complicated the management of migraine and its triggers may be [34,39]. Not coincidentally, respondents who acknowledged a greater complexity in the management of migraine not only had frequently characterized migraine as a common (aOR 2.730; 95%CI 1.495 to 4.984) and severe (aOR 2.347; 95%CI 1.277 to 4.311) condition, but had previous experience in the management of conditional fitness for migraineur workers (aOR 4.761, 95%CI 1.781 to 12.726). Not coincidentally, having received previous requests for medical surveillance from migraineur workers was identified as a main effector for specific requirements (aOR 22.878; 95%CI 4.816; 108.683).

A possible explanation of these results may therefore relate to the impact of personal experiences. Attitudes formed through direct experience with the actual object of the attitude have been found to effectively predict later behaviors [44]; more precisely, individual experience with the assessed topic usually represents the main predictor for the likelihood that the person will properly cope with that behavior [45,46].

However, this explanation possibly represents an oversimplification. First, all cumulative scores (GKS, RPS, number of prescribed interventions, acknowledged difficulties in the managing of migraine) were substantially comparable between professionals having previously managed or not migraineur workers, undermining the role of professional experience. Second, while European and Italian legal frameworks for occupational health and safety prioritize general interventions over individual ones [37,47], being an OP with a specific qualification in occupational medicine was a strong effector of prescribing individual interventions to migraineur workers (aOR 20.326; 95%CI 2.642 to 156.358). On the other hand, OP operating in healthcare settings from the National Health Service were less likely to meet specific individual requirements, even though healthcare workers should be otherwise acknowledged at high risk for migraine relapses (aOR 0.036; 95%CI 0.006 to 0.205) [48,49]. In other words, main effectors of reported practices were only partially consistent with identifying personal experience as the main driver for the managing of migraineur workers (See Appendix A Table A1). While having experienced substantial difficulties in managing migraineur workers may have led participating OP to raise their concern towards this disorder, a clear causal relation cannot therefore be inferred. As previous training and having participated into specific informative interventions on migraine had no substantial effect on promoting an increased risk perception or leading towards more extensive prescriptions, our data stress how difficult it may be to bring the attention of professionals towards a more accurate managing of migraine in the workplace.

*Limits of this study*. Despite its novelty and its potential significance, our study is affected by several limits. Firstly, it shares all limits of Internet-based surveys [21,50,51], and mostly the extensive “self-selection” of participants. In similar studies, certain sub-groups may be largely oversampled, impairing the overall reliability of collected results, and in particular: subjects familiar in sharing personal information through internet and social media; individuals exhibiting a proactive attitude or greater knowledge about the assessed topic, etc. Similarly, the fact of not participating could be understood as a negative attitude or a lack of knowledge about the targeted topic [50]. In this regard, the potential self-selection of the participants may have been somewhat mitigated by targeting a very specific and therefore quite homogenous subgroup of medical professionals, i.e., OP.

Second, our sample was based on a small, convenience study group of 242 OP, i.e., 12.1% of the eligible population, but also 3.2% of all officially registered Italian OP (*n* = 7722 by 19 January 2022), which could be hardly considered fully representative of the national level. In fact, assuming as reference the prevalence of migraine in the Italian population (i.e., 20% to 21% [14,28]), an I error of 5% (0.05), and a power of 95%, a minimum sample size may be calculated as follows:

1.96^2^ × 0.705 × (1 − 0.705)/0.05^2^ = 3.8416 × 0.67 × 0.33/0.0025 = 320.

In other words, the present study was hardy generalizable, particularly in a country, such as Italy, characterized by distinctive regional patterns, also considering school-specific training during the residency program in occupational medicine [37].

Third, we cannot rule out a significant social desirability bias, at least affecting the knowledge test. More precisely, participants would not only reporting “common sense” answers, as previously discussed, but also those answers that may have been perceived as more “appropriate” to fit with the aim of the questionnaire. Social desirability bias is quite common in KAP studies, and was specifically addressed in studies focusing on OP [19,20,52,53]. Therefore, we cannot rule out that our results could also have ultimately overstated the share of individuals with an effective understanding of migraine.

Fourth, even though discussion groups (e.g., by registering only subjects who receive a specific invitation by the manager; answering to specific “selection” questions; etc.) involved in the recruitment of the study participants usually perform a preventive selection, we cannot rule out that some of the respondents did not fully adhere to our selection criteria, further compromising the actual representativity of the sample.

Despite the aforementioned limits, our study advocates a stronger collaboration between OP and professionals involved in the management of migraine in the general population [9,15]. In this regard, our methodology could be implemented in future studies in order to assess a broader array of neurological disorders [5], and particularly headache [9], whose overall burden deeply affects the quality of life of affected individuals, creating a long-lasting imbalance between occupational requirements and workers’ potential performances [9,36,54]. Finally, future studies should also aim to distinctively evaluate a broader array of different work settings, focusing on both specific risk factors and properly tailored preventive interventions.

## 5. Conclusions

In conclusion, migraine is a common disorder, which represents a challenging clinical problem for OP. In this convenience sample, participating OP exhibited a substantial understanding of migraine and its triggers, but residual false beliefs and common misunderstanding may impair the proper management of this disorder. While it is reasonable that personal experience with problematical cases among migraineur workers and the understanding of frequency and clinical relevance of migraine may have led to a better understanding of migraine as a complicated disorder to manage in the workplace, our results are somewhat conflicting, suggesting a more complicated process of decision making towards an appropriate management of migraine. While new therapeutic options for migraine are made available, innovative and more specifically tailored formation of OP, even providing specific skills from other medical branches during residency, may increase their capability to cope with the requirements of migraineur workers in a cost-effective way. Therefore, more extensive research on the management of migraine in the workplaces are highly required.

## Figures and Tables

**Table 1:** Characteristics of the 242 Italian occupational physicians (OP) participating into the survey on knowledge, attitudes and practices on migraine in the workplaces.

Variable	No./242, %	Average ± SD
Gender		
Male	163, 67.4%	
Female	79, 32.6%	
Age (years)		47.8 ± 8.8
Age ≥ 50 years	85, 35.1%	
Seniority as OP		21.1 ± 13.7
Seniority ≥ 10 years	184, 76.0%	
Italian region		
Northern Italy ^1^	110, 45.5%	
Central Italy ^2^	69, 28.5%	
Southern Italy ^3^	55, 22.7%	
Undisclosed	8, 3.3%	
Qualification as OP		
Specialization in occupational medicine	215, 88.8%	
Specialization in hygiene and preventive medicine	6, 2.5%	
Specialization in legal medicine	13, 5.4%	
Other authorizations	6, 2.5%	
Working as OP in Hospital(s) affiliated with National Health Service	42, 17.2%	
Any basic formation (at least 6 months during residency)		
Neurology	34, 14.0%	
Psychiatry	11, 4.5%	
Internal medicine	156, 64.5%	
Information sources		
Professional courses	194, 81.7%	
Medical journals	124, 51.2%	
Books	91, 37.6%	
Colleagues	106, 43.8%	
Official websites	150, 62.0%	
New Media (blog, social media, wikis, etc.)	37, 15.3%	
Any previous course on migraine	20, 8.5%	
Acknowledging migraine as a severe disorder	155, 60.0%	
Acknowledging migraine as a common disorder	127, 54.0%	
General Knowledge Score		74.0% ± 14.3
General Knowledge Score > median value (72.7%)	98, 40.5%	
Risk Perception Score		54.1% ± 18.7

^1^ Aosta Valley, Piedmont, Liguria, Lombardy, Veneto, Autonomous Province of Trento, Autonomous Province of Bolzano, Friuli-Venezia-Giulia, Emilia Romagna; ^2^ Tuscany, Umbria, Marche, Lazio ^3^ Campania, Abruzzo, Apulia, Basilicata, Calabria, Sicily, Sardinia.

**Table 2:** Knowledge test: response distribution of presented items proposed to the 242 medical professionals participating in the survey and contributing to the assessment of general knowledge score (GKS) (Cronbach’s alpha = 0.744).

Statement	Correct Answer	Total (No./242)
Migraine usually affects 1 out of 3 individuals of female gender	TRUE	138, 58.7%
Clinically, headache associated with migraine is usually bilateral and pulsating	FALSE	152, 64.7%
Relapses of migraine may last between 4 and 72 h	TRUE	206, 87.7%
Migraine has emotional, cognitive and behavioral features	TRUE	229, 97.4%
In females, migraine usually results in a better quality of life than in males	FALSE	164, 69.8%
Males affected by migraine are usually affected by greater loss of productivity	TRUE	125, 53.2%
Females exhibit greater presenteeism despite pain and malaise	TRUE	191, 81.3%
In Italy, the majority of affected cases receive appropriate preventive treatment	FALSE	166, 70.6%
Stress and hormonal imbalance represent risk factors for relapses	TRUE	228, 97.0%
Noise and bright light can trigger relapses of migraine	TRUE	230, 97.9%
Extreme low or high temperatures do not represent triggers for migraine relapses	FALSE	86, 36.6%

**Table 3:** Attitudes on the management of migraine in the workplaces from 242 Italian occupational physicians (OP) participating into the survey.

Variable	Total (No./242, %)
Perceived barriers for proper managing of migraineur workers	
Ergonomics	78, 33.2%
Intervention on work-related risk factors for migraine	73, 31.1%
Intervention on individual risk factors for migraine	112, 47.7%
Working hours	116, 49.4%
Work rhythms	124, 52.8%
Work-related stress	144, 61.3%
Any previous interaction with migraineur workers	159, 65.7%
Previously planned specific medical surveillance for migraineur workers (any)	12, 5.0%
Any request of medical surveillance from a migraineur worker	89, 37.9%
Last year	50, 20.7%
Last 5 years	86, 35.5%
Diagnosis of migraine following occupational assessment	43, 17.8%
Previously judged workers “conditionally fit” because of migraine	132, 54.5%
Medical requirements in conditional fitness	
… avoiding night shifts	69, 28.5%
… avoiding shiftwork	30, 12.4%
… avoiding exposures to extreme temperatures	32, 13.2%
… avoiding exposures to extreme intense lights	50, 20.7%
… avoiding front-office activities	44, 18.2%
… avoiding exposures to irritating chemical agents	9, 3.7%
… increased number/length of pauses	49, 20.2%
Previously judged workers “unfit” because of migraine	20, 8.3%
Previously received any appeal for medical judgement of fitness/unfitness	7, 2.9%

**Table 4:** Perceived difficulty in the managing of migraine in the workplaces as reported by participants through a synthetic score 1 (no concern) to 10 (very high concern), compared to a series of common disorders. Kruskal–Wallis rank sum test was performed in order to compare migraine (assumed as a reference category) to the other disorders. Perceived concern score was then dichotomized in low concern (i.e., 1 to 5) vs. high concern (6 to 10).

Perceived Difficulty of the Managing in the Workplaces	Score (1–10) Average ± SD	Kruskal–Wallis Rank Sum *p*-Value	High Concern (Score > 5) No/242, %
Migraine	6.0 ± 2.0	REFERENCE	148, 61.2%
Diabetes	6.3 ± 1.8	0.338	160, 66.1%
Asthma	6.2 ± 1.9	0.525	161, 66.5%
Low back pain	7.2 ± 2.1	<0.001	189, 78.1%
Work-related upper arm disorders	7.1 ± 1.9	<0.001	192, 79.3%
Chronic heart disease	7.4 ± 1.6	<0.001	208, 86.0%
Fibromyalgia	6.9 ± 2.3	<0.001	184, 76.0%
Depression	7.2 ± 1.9	<0.001	198, 81.8%
Epilepsy	6.8 ± 2.3	<0.001	188, 77.7%

**Table 5:** Analysis of factors that in participating Italian occupational physicians (No. = 242) were associated with greater perceived difficulty in the managing of migraine in the workplaces. Comparisons were performed by means of chi squared test. All factors that, in univariate analysis, were associated with the outcome variable of higher concern regarding the managing of migraine (*p* < 0.050) were included a logistic regression analysis model as explanatory variables, with calculation of corresponding adjusted odds ratios (aOR) and their respective 95% confidence intervals (95%CI).

Variable	Perceived Difficulty in the Managing of Migraine in the Workplaces		*p*-Value	aOR (95%CI)
	High Concern (No./148, %)	Low Concern (No./94, %)		
Male Gender	48, 32.4%	31, 33.0%	0.930	-
Age ≥ 50 years	50, 33.8%	35, 37.2%	0.584	-
Seniority ≥ 10 years	113, 76.4%	71, 75.5%	0.884	-
Operating in Northern Italy	69, 46.6%	41, 43.6%	0.647	-
Specialization in occupational medicine	134, 90.5%	87, 92.6%	0.588	-
Working as OP in Hospital(s) affiliated with the National Health Service	26, 17.6%	16, 17.0%	1.000	-
Previous training in neurology	15, 10.5%	19, 20.2%	0.037	0.703 (0.244; 2.028)
Any previous course on migraine	13, 8.9%	9, 9.6%	0.861	-
GKS ≥ median value (72.7%)	62, 41.9%	36, 38.3%	0.674	-
Information sources				
Professional courses	116, 78.4%	78, 83.0%	0.478	-
Medical journals	79, 53.4%	45, 47.9%	0.482	-
Books	53, 35.8%	38, 40.4%	0.558	-
Colleagues	49, 33.1%	57, 60.6%	<0.001	0.206 (0.091; 0.466)
Official websites	90, 60.8%	60, 63.8%	0.737	-
New Media (blog, social media, wikis, etc.)	19, 12.8%	18, 19.1%	0.252	-
Migraine acknowledged as …				
… a frequent/very frequent disorder	94, 63.5%	37, 39.4%	<0.001	3.672 (1.526; 8.833)
… a severe/very severe disorder	112, 75.7%	47, 50.0%	<0.001	1.878 (0.809; 4.356)
Any previous interaction with MW	99, 67.8%	60, 63.8%	0.525	-
Planned medical surveillance for MW	6, 4.1%	6, 6.4%	0.416	-
Any request of medical surveillance from MW	62, 41.9%	32, 34.0%	0.222	1.043 (0.447; 2.432)
Any diagnosis of migraine in medical practice	30, 20.3%	13, 13.8%	0.201	0.872 (0.306; 2.484)
Any previous judgement of “conditional fitness” because of migraine	94, 62.8%	39, 41.5%	0.001	4.761 (1.781; 12.726)
Any previous judgement of “unfitness” because of migraine	17, 11.5%	4, 4.3%	0.051	3.599 (0.919; 14.097)
Any appeal for medical judgement of fitness/unfitness	5, 3.5%	2, 2.1%	0.543	-

Note: GKS = General Knowledge Score; MW = migraineur workers.

**Table 6:** Analysis of factors that in participating Italian occupational physicians (OP) having previously managed migraineur workers (MW, No. = 159) were associated with having applied any conditional medical judgment and/or restriction. Comparisons were performed by means of chi squared test. All factors that in univariate analysis were associated with the outcome variable of having reported at least an intervention for MW (*p* < 0.050), were included a logistic regression analysis model as explanatory variables, with calculation of corresponding adjusted odds ratios (aOR) and their respective 95% confidence intervals (95%CI).

Variable	Reported Interventions for MWs		*p*-Value	aOR (95%CI)
	At least 1 (No./121, %)	None (No./38, %)		
Male Gender	38, 31.4%	13, 34.2%	0.901	-
Age ≥ 50 years	76, 62.8%	24, 63.2%	1.000	-
Seniority ≥ 10 years	104, 86.0%	28, 73.7%	0.131	-
Operating in Northern Italy	53, 43.8%	15, 39.5%	0.778	-
Specialization in occupational medicine	116, 95.9%	31, 81.6%	0.011	20.326 (2.642; 156.358)
Working as OP in Hospital(s) affiliated with the National Health Service	11, 9.1%	13, 34.2%	<0.001	0.036 (0.006; 0.205)
Previous training in neurology	17, 14.5%	6, 15.8%	1.000	-
Any previous course on migraine	18, 14.9%	2, 5.3%	0.201	-
GKS ≥ median value (72.7%)	43, 44.6%	15, 39.5%	0.710	-
Information sources				-
Professional courses	98, 81.0%	31, 81.6%	1.000	-
Medical journals	68, 56.2%	17, 44.7%	0.294	-
Books	41, 33.9%	14, 36.8%	0.890	-
Colleagues	52, 43.0%	18, 47.4%	0.773	-
Official websites	75, 62.0%	29, 76.3%	0.154	-
New Media (blog, social media, wikis, etc.)	18, 14.9%	7, 18.4%	0.788	-
Migraine acknowledged as …				
… a frequent/very frequent disorder	66, 54.5%	26, 68.4%	0.186	-
… a severe/very severe disorder	86, 71.1%	24, 63.2%	0.471	-
… difficult to manage in the workplaces	83, 68.6%	16, 42.1%	0.006	2.715 (1.034; 7.128)
Planned medical surveillance for MW	12, 9.9%	0, -	0.096	-
Any request of medical surveillance from MW	78, 64.5%	6, 15.8%	<0.001	22.878 (4.816; 108.683)
Any diagnosis of migraine in medical practice	32, 26.4%	3, 7.9%	0.029	1.804 (0.399; 8.164)
Any appeal for medical judgement of fitness/unfitness	4, 3.4%	0, -	0.576	-

Note = GKS, General Knowledge Score.

## Data Availability

The data presented in this study are available on request from the corresponding author.

## Appendix A

**Table A1:** Association of main variables with having managed migraineur workers in occupational practice by 242 occupational physicians (OP) participating into the analyses (two of them not replying to the item) (univariate analysis, chi quadred test).

Variable	Having Previously Managed Migraineur Workers		*p*-Value
	ANY (No./159, %)	NEVER (No./81, %)	
Male Gender	51, 32.1%	38, 34.6%	0.808
Age ≥ 50 years	59, 37.1%	26, 32.1%	0.532
Seniority ≥ 10 years	132, 83.0%	50, 61.7%	<0.001
Operating in Northern Italy	68, 42.8%	40, 49.4%	0.403
Specialization in occupational medicine	147, 92.5%	74, 91.4%	0.965
Working as OP in Hospital(s) affiliated with the National Health Service	24, 15.1%	18, 22.2%	0.232
Previous training in neurology	23, 14.8%	11, 13.8%	0.977
Any previous course on migraine	20, 12.6%	2, 2.5%	0.022
GKS ≥ median value (72.7%)	69, 43.4%	29, 35.8%	0.321
Information sources			
Professional courses	129, 81.1%	63, 77.8%	0.657
Medical journals	85, 53.5%	37, 45.7%	0.316
Books	55, 34.6%	34, 42.0%	0.328
Colleagues	70, 44.0%	36, 44.4%	1.000
Official websites	104, 65.4%	46, 56.8%	0.245
New Media (blog, social media, wikis, etc.)	25, 15.7%	12, 14.8%	1.000
Migraine acknowledged as …			
… a frequent/very frequent disorder	92, 57.9%	39, 48.1%	0.196
… a severe/very severe disorder	110, 69.2%	49, 60.5%	0.229
… difficult to manage in the workplaces	99, 62.3%	47, 58.0%	0.620
Planned medical surveillance for MW	12, 7.5%	0, -	0.026
Perceived barriers for proper managing of migraineur workers			
Ergonomics	53, 33.3%	28, 34.6%	0.963
Intervention on work-related risk factors for migraine	47, 29.6%	30, 37.0%	0.304
Intervention on individual risk factors for migraine	91, 57.2%	28, 34.6%	0.001
Working hours	89, 56.0%	38, 46.9%	0.233
Work rhythms	53, 33.3%	35, 43.2%	0.174
Work-related stress	101, 63.5%	45, 55.6%	0.291

Note: GKS, general knowledge score; MW, migraineur workers.
